# A Report of Brugada Syndrome Presenting with Cardiac Arrest Triggered by Verapamil Intoxication

**DOI:** 10.4274/balkanmedj.2016.1301

**Published:** 2017-12-01

**Authors:** Kahraman Yakut, İlkay Erdoğan, Birgül Varan, İlyas Atar

**Affiliations:** 1 Department of Pediatric Cardiology, Başkent University Ankara Hospital, Ankara, Turkey; 2 Department of Cardiology, Başkent University Ankara Hospital, Ankara, Turkey

**Keywords:** Brugada syndrome, sudden death, cardiac arrhythmia, diagnosis

## Abstract

**Background:**

Brugada syndrome is a disease characterized by a specific electrocardiographic pattern and an increased risk of sudden cardiac death. We present this case with the updated literature to emphasise the need to consider the diagnosis of Brugada syndrome in patients admitted to the emergency ward with sudden cardiac arrest.

**Case Report:**

A 16-year-old female patient was admitted to the emergency ward with complaints of weakness and abdominal pain, and she had four cardiac arrests during her evaluation period. She was referred to our clinic for permanent pacemaker implantation. She was on a temporary pace maker after having had C-reactive protein. Her physical exam was normal except for bilaterally decreased lung sounds. Lung x-ray and computed tomography, which were performed by another institution, revealed minimal pleural effusion and nothing else of significance. Blood and peritoneal fluid samples were sterile. Echocardiographic exam and cardiac enzymes were also in the normal ranges. Electrocardiographic showed incomplete right branch block in leads V1 and V2. An ajmaline test revealed specific electrocardiographic findings of the type I Brugada pattern. We proposed implanting an implantable cardioverter defibrillator to the patient as there were positive findings on the ajmaline test as well as a history of sudden cardiac arrest. After this treatment proposal, the patient’s family admitted that she had taken a high dose of verapamil and thus, the encountered bradycardia was associated with verapamil overuse. The ajmaline test was repeated as it was contemplated that the previous positive ajmaline test had been associated with verapamil overuse. Implantable cardioverter defibrillator implantation was proposed again as there was a history of sudden cardiac arrest; however, the family did not consent to implantable cardioverter defibrillator, and the patient was discharged and followed up.

**Conclusion:**

Brugada syndrome should be considered for patients who are admitted to the emergency ward with sudden cardiac arrest though surface electrocardiographic is normal. If there is a suspicion of Brugada syndrome, repeated electrocardiographic should be performed on different occasions. Diagnosis can be clarified by upper costal electrocardiographic or by administering Na channel blockers during electrocardiographic performance.

Brugada syndrome (BrS) is a hereditary arrhythmia characterized by an electrocardiographic (ECG) pattern and an increased risk of sudden cardiac death without structural abnormalities. The syndrome predominantly affects young adult men, and the first clinical symptoms occur at the age of approximately 40 years (1). We aim to share the case of a 16-year-old patient who was admitted after a resuscitated cardiac arrest and was diagnosed with BrS. It was learned that she had attempted suicide by verapamil overdose before the cardiac arrest.

## CASE PRESENTATION

**FIG. 1.**
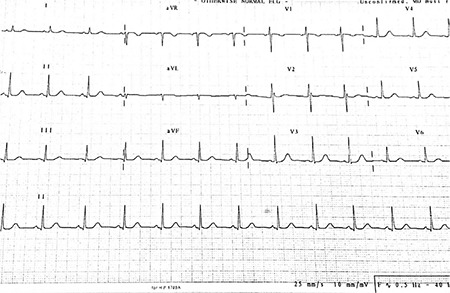
The baseline electrocardiographic examination showed an incomplete right bundle branch block in V1-V2.

**FIG. 2. a, b.**
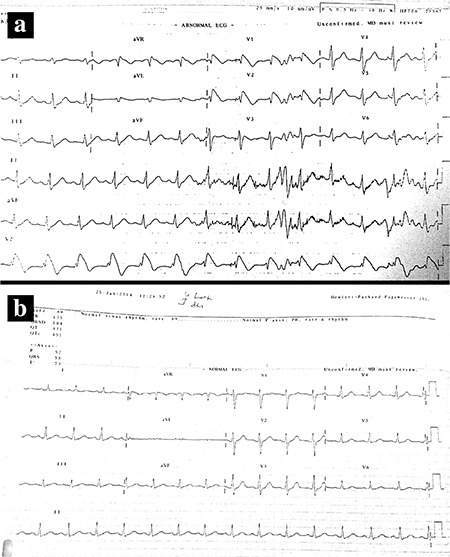
Electrocardiography showed typical Brugada syndrome findings in the third costal position during the ajmaline test, however simultaneus electrocardiographic in the fourth costal position was completely normal.

A 16-year-old girl was admitted to another centre with dizziness, nausea and abdominal pain. Abdominal ultrasonography revealed free fluid in the abdomen. The patient underwent laparoscopy with the suspected diagnosis of ovarian torsion, and diagnostic fluid was drained. She had four episodes of cardiac arrest (asystole) following severe bradycardia during and after this procedure, and a temporary pacemaker was inserted. The patient was referred to our clinic under emergency conditions for permanent pacemaker insertion. The physical examination in our clinic was normal, except for decreased bilateral breath sounds in the lower zones. When the patient and family were insistently questioned regarding intoxication, no information was obtained on this subject. The chest radiogram revealed bilateral minimal pleural effusion and the thoracic tomography demonstrated minimal pleural effusion in both hemithoraces. The blood levels of the acute-phase reactants were normal, blood cultures were negative and rheumatologic investigations were normal. The effusion disappeared with antibiotic treatment for pneumonia during follow-up. The echocardiographic examination, which was performed to exclude myocarditis due to the history of unexplained sudden cardiac arrest, and the cardiac enzymes were normal. The 24-hour Holter monitorization revealed rare premature ventricular contraction. ECG examination showed an incomplete right bundle branch block (RBBB)-like pattern on V1-V2 (Figure 1). No pathology was detected on selective coronary angiography, which was performed to exclude coronary artery abnormalities. Third costal position ECG, which has a higher sensitivity in unmasking type I BrS ECG was used 2). Electrocardiogram showed typical BrS findings in the third costal position during the ajmaline test; however, simultaneous ECG in the fourth costal position was completely normal (Figure 2a, b). Because of the history of cardiac arrest and the positive ajmaline test, implantable cardioverter defibrillator (ICD) implantation was recommended to the patient. The bradycardia and cardiac arrest were attributed to verapamil; however, the positivity of the ajmaline test could not be explained by this, and the test was repeated after the patient had recovered from bradycardia, producing another positive result. ICD insertion was recommended for the development of cardiac arrest (asystole) with bradycardia in our patient. When we insisted on ICD insertion, it was revealed that the patient had attempted suicide by taking a high dose of verapamil. The patient's cardiac arrest was attributed to verapamil intoxication. BrS was diagnosed incidentally in this patient. There was no history of sudden cardiac death and no evidence of BrS on ECGs and genetic screening tests performed on the patient’s family. ICD insertion was not performed because of asymptomatic BrS; the patient was discharged, and follow-up was continued. Using the DNA sample from the patient’s peripheral blood, the presence of c.87A>G (p.A29A) (heterozygous), c.3183A>G (p.E1061E) (heterozygous) and c.5457C>T (p.D1819D) (heterozygous) was identified in the DNA sequence analysis of the SCN5A gene. The patient and her family were provided with information about this current work, and informed consent forms were signed.

## DISCUSSION

BrS is a disease characterized by sudden cardiac death, typically occurring during sleep in relatively young individuals. It may be detected in every age group from early infancy to the elderly, occurring most frequently in the fourth decade of life and the male gender. The primary mechanism of BrS is mutations in the SCN5A gene, which are responsible for regulation of the alpha subunit of sodium channels in the cell membrane of cardiac muscle (3). BrS is characterized by the RBBB pattern and coved-type ST elevation >2 mm in V1-V3 derivations on ECG (1). This ECG pattern is called type I. In some cases, the RBBB pattern and an ST elevation >2 mm with the saddle-back type instead of the coved type were detected, and this ECG pattern is called type II. If it is either a saddle-back or a coved-type ST elevation and <2 mm, it is called type III. Among these ECG patterns, type I is diagnostic, whereas types II and III are not. Additionally, sometimes BrS is hidden with very mild ECG changes. If there are suspicious clinical conditions and syncope with suspected ECGs or unexplained sudden deaths in family members, special tests should be performed to investigate the presence of BrS. These tests are performed by administering sodium channel blockers to patients, such as flecainide, ajmaline, procainamide, propafenone and disopyramide, while recording the changes in the ECG. If the ECG changes show an alteration from types II or III to type I, then BrS should be considered. Since dangerous arrhythmias may develop during these tests, the patient’s rhythm should be monitored closely. For our patient, the diagnosis was confirmed by the positive ajmaline test. The mean age at which symptoms first appear in affected individuals is the third to fourth decade. Among the initial reports however, one described symptomatic twins who were 1 year of age, and more recent reports describe families with symptomatic children (4). It was important in the current case that the patient was a 16-year-old girl, and there was no BrS diagnosis and/or sudden death at a young age in the family history. The reason for the early age of the diagnosis in this patient was her admission with severe bradycardia and cardiac arrest due to verapamil intoxication. In most reported cases, a large number of sudden cardiac deaths at a young age are reported in the family history. The basal ECGs from the screening of the family of the present case and the ajmaline test administered to her parents were evaluated to be negative. The early detection of the initial case is important for diagnosing further secondary cases in the family.

The most common mutations associated with BrS are GPD1L (prevalence of 11%-12%) and SCN5A (prevalence of 18%-30%) mutations (5). To date, approximately 300 mutations in the SCN5A gene have been described in association with BrS. Six other genes (CACNA1C, CACNB2, SCN1B, KCNQ3, SCN3B, and HCN4) have been associated with BrS, but the prevalence of variants in these genes is yet unknown (6). Using the DNA sample from the peripheral blood of the patient, the presence of c.87A>G (p.A29A) (heterozygous), c.3183A>G (p.E1061E) (heterozygous) and c.5457C>T (p.D1819D) (heterozygous) were identified in the DNA sequence analysis of the SCN5A gene. The mutation screening test program was used to investigate the c.5457C>T nucleotide change; however, no association with a disease was established. Additionally, it can be said that there was no change at the level of amino acids when considered for the proteins (p.D1819D) (i.e., this is a silent type of mutation), and this mutation will not cause any specific disease due to not create a difference in the amino acid chain. A previous study reported that the c.5457C>T change was common in the patients with “short QT interval” syndrome; however, this was not reported as directly associated with the disease (7). We believe that the role of such silent mutations in BrS will be much clearer as the number of the cases increase.

In risk stratification and corroboration diagnosis, electrophysiological studies may be useful. According to recent reports, sudden death from ventricular fibrillation (VF) is the first symptom with BrS patients. The episodes of syncope are caused by rapid polymorphic ventricular tachycardia. Approximately 80% of patients with documented VF have a history of syncope (8). Cardiac arrest in patients with BrS generally occurs during sleep or rest (9). Neither ventricular tachycardia (VT) nor VF was induced by EPS in our patient. The single proven effective method of treatment for BrS is the insertion of an ICD (10). Pharmacological treatment approaches have been developed for patients with an ICD inserted and who are exposed to multiple shocks due to arrhythmia attacks (10). Quinidine is suggested as an alternative to ICD for children who have had ICD and who are exposed to many shocks and ventricular arrhythmia storm in particular (10). It is very difficult, especially for paediatric patients, to be convinced about ICD insertion and to adapt themselves to this situation. ICD insertion is not recommended in asymptomatic BrS patients with a drug-induced type I ECG (10). ICD insertion was not performed because of asymptomatic BrS in this case, and the patient continued to be followed up.

BrS can be easily diagnosed by simple ECG changes and medical history; however, it is frequently missed or misdiagnosed in daily practice. BrS should be considered in the differential diagnosis of patients admitted to the emergency service with ST elevation in V1-V3 derivations on ECG, VT, syncope or sudden cardiac arrest. Although there are several technologically advanced diagnostic tools, they are not available in the emergency clinic; however, a basic diagnostic tool like ECG is easily accessible. Given the possibility of sudden cardiac death from BrS, emergency room physicians must be familiar with BrS. Our case also demonstrates once again that risk stratification in the appropriate management of BrS patients is of paramount importance in order to prevent overtreatment with high-risk, life-long cardiac devices.
